# Predictors of mortality in HIV-infected patients starting antiretroviral therapy in a rural hospital in Tanzania

**DOI:** 10.1186/1471-2334-8-52

**Published:** 2008-04-22

**Authors:** Asgeir Johannessen, Ezra Naman, Bernard J Ngowi, Leiv Sandvik, Mecky I Matee, Henry E Aglen, Svein G Gundersen, Johan N Bruun

**Affiliations:** 1 Department of Infectious Diseases, Ulleval University Hospital, Oslo, Norway; 2 HIV Care and Treatment Centre, Haydom Lutheran Hospital, Mbulu, Tanzania; 3 Centre for International Health, University of Bergen, Bergen, Norway; 4 Centre for Clinical Research, Ulleval University Hospital, Oslo, Norway; 5 Department of Microbiology and Immunology, Muhimbili University of Health and Allied Sciences, Dar es Salaam, Tanzania; 6 Research Unit, Sorlandet Hospital HF, University of Agder, Kristiansand, Norway; 7 Faculty for Health and Sports, University of Agder, Kristiansand, Norway

## Abstract

**Background:**

Studies of antiretroviral therapy (ART) programs in Africa have shown high initial mortality. Factors contributing to this high mortality are poorly described. The aim of the present study was to assess mortality and to identify predictors of mortality in HIV-infected patients starting ART in a rural hospital in Tanzania.

**Methods:**

This was a cohort study of 320 treatment-naïve adults who started ART between October 2003 and November 2006. Reliable CD4 cell counts were not available, thus ART initiation was based on clinical criteria in accordance with WHO and Tanzanian guidelines. Kaplan-Meier models were used to estimate mortality and Cox proportional hazards models to identify predictors of mortality.

**Results:**

Patients were followed for a median of 10.9 months (IQR 2.9–19.5). Overall, 95 patients died, among whom 59 died within 3 months of starting ART. Estimated mortality was 19.2, 29.0 and 40.7% at 3, 12 and 36 months, respectively. Independent predictors of mortality were severe anemia (hemoglobin <8 g/dL; adjusted hazard ratio [AHR] 9.20; 95% CI 2.05–41.3), moderate anemia (hemoglobin 8–9.9 g/dL; AHR 7.50; 95% CI 1.77–31.9), thrombocytopenia (platelet count <150 × 10^9^/L; AHR 2.30; 95% CI 1.33–3.99) and severe malnutrition (body mass index <16 kg/m^2^; AHR 2.12; 95% CI 1.06–4.24). Estimated one year mortality was 55.2% in patients with severe anemia, compared to 3.7% in patients without anemia (*P *< 0.001).

**Conclusion:**

Mortality was found to be high, with the majority of deaths occurring within 3 months of starting ART. Anemia, thrombocytopenia and severe malnutrition were strong independent predictors of mortality. A prognostic model based on hemoglobin level appears to be a useful tool for initial risk assessment in resource-limited settings.

## Background

The introduction of highly active antiretroviral therapy in 1996 dramatically improved the prognosis for HIV-infected patients in the industrialized world [1,2]. Until recently, however, access to treatment has been severely limited in developing countries, where the majority of people with HIV/AIDS live [3]. In 2002, the World Health Organization (WHO) issued guidelines for scaling up antiretroviral therapy (ART) in resource-limited settings, followed by revisions in 2003 and 2006 advocating earlier initiation of treatment [4-6]. By December 2006, two million people in low- and middle-income countries were receiving ART, but this was still only 28% of those estimated to be in urgent need of it [7].

Few studies have examined the effect of ART in rural Africa, and experiences from Europe and North America are not necessarily applicable to such settings. However, early reports from ART programs in resource-limited settings have been promising, with virological efficacy comparable to industrialized countries [3]. Nevertheless, mortality has been high, particularly the first months after initiating ART [8-15], and factors contributing to this high mortality are poorly understood.

A better knowledge of prognostic factors would allow closer follow-up and more targeted interventions in high-risk patients, thus reducing excess mortality. The aim of the present study was to assess mortality and to identify predictors of mortality in HIV-infected patients starting ART in a rural African hospital.

## Methods

### Study setting and participants

Tanzania is a low-income country in East Africa with 38.3 million inhabitants and estimated adult HIV prevalence at 6.5% [7]. Life expectancy at birth is 46.5 years, which is estimated to be ten years lower than it would have been without the HIV epidemic [16]. Haydom Lutheran Hospital is a 400-bed hospital in Manyara region owned by the Evangelical Lutheran Church of Tanzania. It is the main health care provider to a rural population of about 260 000 people, and available services include a modern radiology department with ultrasonography and computer tomography, a fairly well equipped laboratory with microscopy, bacteriology and biochemistry, as well as standard surgical and obstetrical services. According to a recent population-based survey, adult HIV prevalence in the area is 1.8% [17]. In 2002, the hospital launched a comprehensive HIV prevention and intervention program with emphasis on voluntary counseling and testing (VCT) through outreach services and antenatal clinics. An HIV Care and Treatment Centre was established adjacent to the hospital, and from October 2003 ART was provided free of charge to eligible HIV-infected patients. Most of the patients enrolled were detected through VCT services in the villages or were hospitalized patients tested on clinical suspicion. Clinical officers, under supervision of a physician, were responsible for medical follow-up of patients. On-site training was provided by HIV specialists from collaborating institutions in Norway. All patients received pre-treatment counselling, and peer-support groups were set up in the major villages. A community home-based care network was established to follow-up adherence and trace missing patients.

Patients were considered eligible for ART if they were in WHO stage IV irrespective of CD4 cell count, WHO stage III with CD4 ≤ 350 cells/μL, or had CD4 ≤ 200 cells/μL regardless of clinical stage, in accordance with WHO and Tanzanian guidelines [5,18]. However, since CD4 cell counts measured by manual techniques were observed to be unreliable, ART initiation was based solely on clinical criteria (WHO stage III and IV) in most patients. In addition, ART was offered to HIV-infected pregnant and lactating women to prevent vertical transmission.

The present study is a prospective, observational cohort study of treatment-naïve patients aged 15 years or older who started ART in Haydom Lutheran Hospital between October 3, 2003, and November 5, 2006. Women who were pregnant at the time of ART initiation were excluded from the study, as were lactating mothers in WHO stage I or II, who started ART exclusively to prevent vertical transmission. Follow-up data was collected through May 5, 2007. Patients gave written consent to participate in the study. Ethical approval was obtained from the Medical Research Coordinating Committee of the National Institute for Medical Research in Tanzania and Regional Committee for Medical Research Ethics in Norway.

### Treatment, monitoring and endpoints

First-line treatment comprised stavudine (d4T) or zidovudine (ZDV), combined with lamivudine (3TC), and either nevirapine (NVP) or efavirenz (EFV). Regimen choice was subject to availability, with use of a generic fixed-dose combination of d4T, 3TC and NVP whenever possible. Second-line treatment in case of treatment failure was not available until December 2006. Patients with CD4 ≤ 200 cells/μL or WHO stage III or IV disease were given co-trimoxazole prophylaxis 960 mg thrice weekly or 480 mg daily. After the initial 2 weeks of daily drug administration, antiretroviral drugs were dispensed on a monthly basis.

A standardized form was used for the baseline evaluation, which included socio-demographic information, medical history, physical examination, and laboratory investigations. Clinical staging was performed using the 2003 revision of the WHO clinical staging system [5]. Routine clinical follow-up was scheduled every 3 months. HIV infection was established using 2 different rapid antibody tests. Standard hematology was measured using Sysmex KX-21 Hematology Analyzer (Sysmex Corp., Kobe, Japan).

The most recent laboratory results before starting ART were generally used as baseline values. In a minority of patients who lacked pre-treatment laboratory tests, however, results obtained within one month of ART initiation were used. If two values were obtained within a month, the mean was employed. Body mass index (BMI, weight in kilograms divided by height in meters squared) was used to assess nutritional status. Body weight was measured at each clinic visit using the same manual scale, and height was measured using a stadiometer mounted on the scale. Established cutoff values for BMI were used [19]: normal (BMI ≥ 18.5 kg/m^2^), mild malnutrition (BMI 17–18.4 kg/m^2^), moderate malnutrition (BMI 16–16.9 kg/m^2^), and severe malnutrition (BMI < 16 kg/m^2^). Anemia was defined as a hemoglobin level of <12 g/dL for women and <13 g/dL for men [20], and was classified as mild (hemoglobin 10–11.9 g/dL for women and 10–12.9 g/dL for men), moderate (hemoglobin 8–9.9 g/dL) or severe (hemoglobin < 8 g/dL). Lymphopenia was defined as a total lymphocyte count (TLC) of <1.2 × 10^9^/L [4], and we employed an additional cutpoint at 0.6 × 10^9^/L to assess severe lymphopenia. Thrombocytopenia was defined as platelet count <150 × 10^9^/L [21].

The main endpoint in our study was death from all causes. Deaths were registered from hospital records or reported through home visitors. Other outcomes were also recorded, including patients who self-stopped treatment, were transferred to another health facility or were lost to follow-up. Patients who missed appointments for more than 3 months and could not be traced by the home visitor, were regarded lost to follow-up.

### Statistical analysis

Patients were excluded from the study if sex, age or WHO stage was not recorded. Date of death was registered by home visitors; however, in 7 patients with only month and year recorded we used the 1^st ^of that month, and in 2 patients with unknown death date we used the last follow-up visit. For subjects who self-stopped treatment, were transferred out or were lost to follow-up, the date of their last follow-up visit was used as the censoring date. Finally, individuals alive and on ART were censored at May 5, 2007.

Kaplan-Meier models were used to estimate survival after ART initiation, and log rank tests to compare survival curves. Cox proportional hazards models were used to identify independent predictors of mortality and calculate hazard ratios. Multicollinearity was excluded using Spearman's correlation coefficient with a cutoff at 0.7. We performed univariable Cox regression analysis for the following baseline variables: sex, age, tribe, religion, education level, ART start year, WHO stage, BMI, hemoglobin, TLC, platelet count, hepatitis B, syphilis and active tuberculosis (TB). CD4 cell counts were omitted since the results were observed to be inaccurate. Baseline variables significant at *P *< 0.05 level in univariable analysis were included in the final multivariable model. We used SPSS version 14.0 software (SPSS Inc., Chicago, IL, USA) to analyze the data. All tests were two-sided and level of significance was set at *P *< 0.05.

## Results

### Baseline characteristics

**Figure 1:**
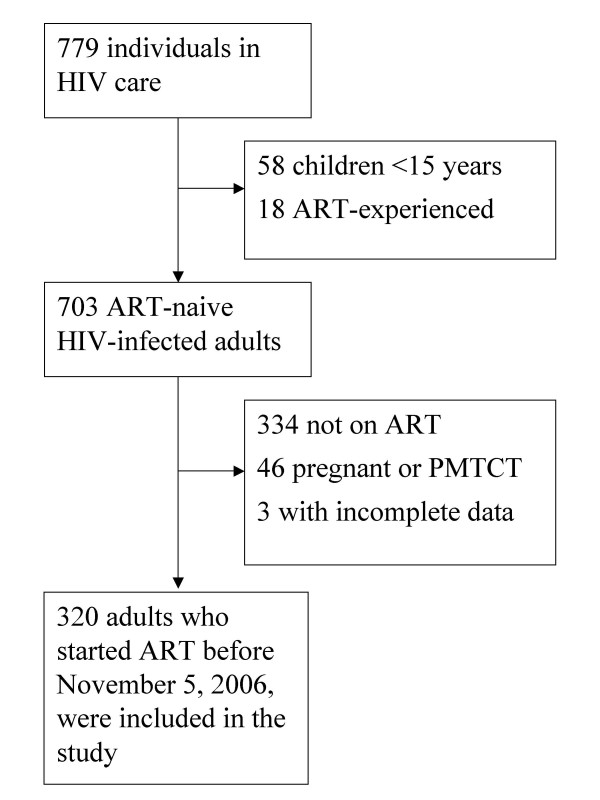
Profile of the study cohort, Haydom Lutheran Hospital, Tanzania (October 2003–November 2006).

Of 779 patients enrolled into HIV care between October 3, 2003, and November 5, 2006, 320 treatment-naïve non-pregnant adults who started ART were included in the present study. The cohort profile is presented in figure 1. Among 334 adults who had not started ART at censoring, 123 (36.8%) were lost to follow-up, 90 (26.9%) did not meet clinical criteria for starting ART, 56 (16.8%) died before ART initiation, 27 (8.1%) were transferred to another health facility, and the remaining 38 (11.4%) were still waiting to start treatment.

Patients on ART were followed for a median of 10.9 months (interquartile range 2.9–19.6). Summary statistics of baseline characteristics are given in table 1. Of the 320 patients included, 223 (69.7%) were women and median age was 35 years (interquartile range 30–43). There were 104 patients (32.5%) who started ART in the initial years 2003–04, 117 (36.6%) started in 2005, and 99 (30.9%) in 2006. Initial ART regimen was d4T/3TC/NVP in 168 patients (52.5%), d4T/3TC/EFV in 58 (18.1%), ZDV/3TC/NVP in 53 (16.6%), ZDV/3TC/EFV in 24 (7.5%), ZDV/3TC/tenofovir in one (0.3%) and missing in 15 patients (4.7%). Seventy-three patients received anti-TB treatment at inclusion or started after inclusion. Mean BMI was 17.6 kg/m^2 ^(standard deviation [SD] 3.1), mean hemoglobin 10.1 g/dL (SD 2.1), mean TLC 1.4 × 10^9^/L (SD 0.8) and mean platelet count 266 × 10^9^/L (SD 131).

**Table 1:** Baseline characteristics and associated mortality among 320 HIV-infected patients starting ART in Tanzania

**Characteristic**	**Number of patients**	**Number of Deaths**
**Age (years)**		
15–24	26	7 (26.9%)
25–34	129	39 (30.2%)
35–44	95	30 (31.6%)
≥ 45	70	19 (27.1%)
**Sex**		
Male	97	38 (39.2%)
Female	223	57 (25.6%)
**Clinical stage**		
WHO stage I–II	12	1 (8.3%)
WHO stage III	98	18 (18.4%)
WHO stage IV	210	76 (36.2%)
**BMI (kg/m**^2^**)**^a^		
<16	98	46 (46.9%)
16–18.4	105	23 (21.9%)
≥ 18.5	93	14 (15.1%)
**Hemoglobin (g/dL)**^b^		
<8	49	27 (55.1%)
8–9.9	108	43 (39.8%)
10–11.9 (10–12.9 for men)	104	21 (20.2%)
≥ 12 (≥ 13 for men)	55	2 (3.6%)
**TLC (× 10**^9^**/L)**^c^		
<0.6	30	18 (60.0%)
0.6–1.1	116	32 (27.6%)
≥ 1.2	166	42 (25.3%)
**Platelet count (× 10**^9^**/L)**^d^		
<150	52	24 (46.2%)
≥ 150	261	66 (25.3%)

^a^24 values missing (n = 296). ^b^4 values missing (n = 316). ^c^8 values missing (n = 312). ^d^7 values missing (n = 313).

WHO, World Health Organization; BMI, body mass index; TLC, total lymphocyte count.

At ART initiation, 210 patients (65.6%) had clinical AIDS (WHO stage IV). For comparison, 401 (51.5%) of 779 had clinical AIDS at enrollment into the HIV program. The most common WHO stage IV conditions among patients who started ART were: wasting syndrome (89.0%), oesophageal candidiasis (13.3%), extrapulmonary TB (5.2%) and Kaposi's sarcoma (4.8%).

### Survival analysis

Overall, 95 patients (29.7%) died during the follow-up period, among whom 59 died within 3 months of starting ART. Thirty-five patients (10.9%) were transferred to another health facility, 31 (9.7%) were lost to follow-up and 7 (2.2%) self-stopped treatment. Estimated mortality was 19.2, 24.5, 29.0, 35.2 and 40.7% at 3, 6, 12, 24 and 36 months, respectively.

In univariable analysis male sex, ART start year, WHO stage IV, severe malnutrition, anemia, lymphopenia and thrombocytopenia were all associated with progression to death. No such associations were found for age, tribe, religion, education level, hepatitis B, syphilis or active TB. As described in table 1, certain baseline values were missing in 29 patients; hence, there were 291 patients in the final Cox model. In multivariable analysis significant predictors of mortality were severe and moderate anemia, thrombocytopenia and severe malnutrition (Table 2). The hazard of death was significantly reduced in those starting ART in calendar year 2006 compared with the initial period 2003–04.

**Table 2:** Hazard ratios of mortality according to baseline variables in HIV-infected patients starting ART in Tanzania

	**Unadjusted**		**Adjusted**^a^	
**Baseline variables**	**HR (95% CI)**	***P***	**HR (95% CI)**	***P***

Gender (male vs. female)	1.73 (1.15–2.61)	0.009	1.60 (1.00–2.57)	0.053
WHO stage (IV vs. I–III)	2.71 (1.64–4.49)	<0.001	1.46 (0.81–2.65)	0.210
ART start year (vs. 2003–04)				
2005	0.55 (0.35–0.87)	0.010	0.64 (0.38–1.08)	0.091
2006	0.30 (0.17–0.56)	<0.001	0.40 (0.19–0.83)	0.014
BMI (vs. ≥ 18.5 kg/m^2^)				
<16	4.17 (2.29–7.60)	<0.001	2.12 (1.06–4.24)	0.034
16–18.4	1.60 (0.82–3.10)	0.168	1.27 (0.62–2.61)	0.516
Hemoglobin (vs. ≥ 12 g/dL for women and ≥ 13 for men)				
<8	22.7 (5.40–95.7)	<0.001	9.20 (2.05–41.3)	0.004
8–9.9	13.5 (3.28–55.9)	<0.001	7.50 (1.77–31.9)	0.006
10–11.9 (10–12.9 for men)	6.21 (1.46–26.5)	0.014	4.03 (0.93–17.5)	0.063
TLC (vs. ≥ 1.2 × 10^9^/L)				
<0.6	3.58 (2.05–6.24)	<0.001	1.72 (0.87–3.39)	0.117
0.6–1.1	1.10 (0.69–1.74)	0.699	0.79 (0.48–1.32)	0.371
Platelet count (<150 vs. ≥ 150 × 10^9^/L)	2.23 (1.40–3.57)	0.001	2.30 (1.33–3.99)	0.003

^a^Cox proportional hazards model adjusted for all variables listed in the table.

HR, hazard ratio; CI, confidence interval; ART, antiretroviral therapy; WHO, World Health Organization; BMI, body mass index; TLC, total lymphocyte count.

**Figure 2:**
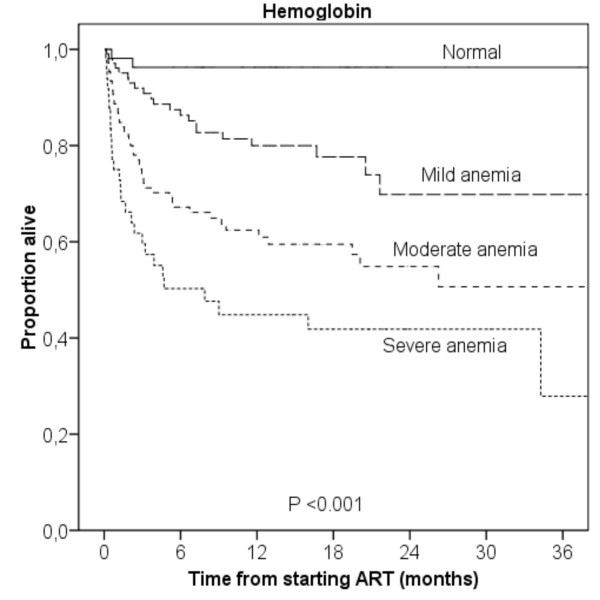
**Kaplan-Meier survival curves according to baseline hemoglobin**. Normal: >12 g/dL (>13 g/dL for men); mild anemia: 10–11.9 g/dL (10–12.9 g/dL for men); moderate anemia: 8–9.9 g/dL; severe anemia: <8 g/dL.

**Figure 3:**
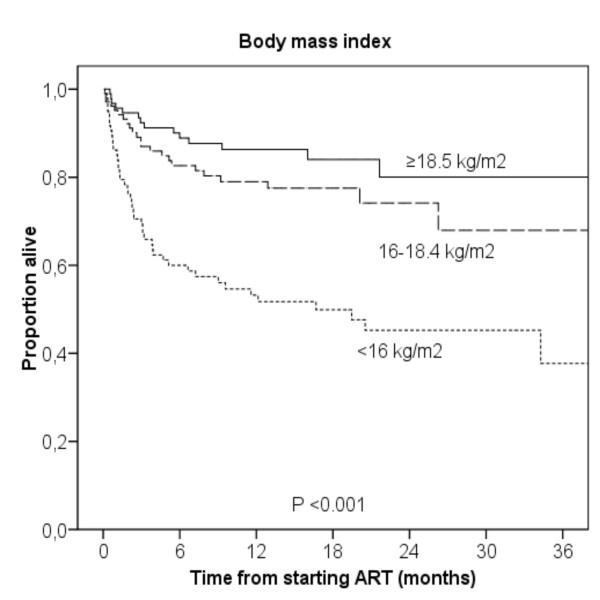
Kaplan-Meier survival curves according to baseline body mass index.

Mortality increased with decreasing hemoglobin. Estimated one year mortality was 3.7% in patients without anemia, 20.0% in mild anemia, 37.6% in moderate anemia and 55.2% in severe anemia (log rank test, *P *< 0.001, Figure 2). The majority of deaths occurred early, and the corresponding 3 months mortality was 3.7, 8.1, 26.9 and 40.4%, respectively (log rank test, *P *< 0.001). A similar trend was observed with decreasing BMI. Estimated one year mortality was 13.7% in patients with normal nutritional status, 21.0% in mild to moderate malnutrition, and 46.8% in severe malnutrition (log rank test, *P *< 0.001, Figure 3).

## Discussion

Mortality was high in this cohort, and most of the deaths occurred within 3 months of starting ART. Severe and moderate anemia, thrombocytopenia and severe malnutrition were found to be independent predictors of mortality. The high early mortality observed in our study is in line with other similar studies from resource-limited settings [8-15]. Causes of death were not investigated in the present study; however, in a study from South Africa wasting syndrome, TB, acute bacterial infections, malignancies and immune reconstitution disease were the major causes of death [14]. In our cohort more than half of the patients had clinical AIDS at enrollment into HIV care, and other African ART programs have also reported high rates of advanced disease [8-12,14,15]. Stigma and delay in seeking health care, lack of voluntary testing and counseling services, and health system delays in referral and ART initiation are possible explanations. Thus, priority must be given to identify HIV-infected individuals and start treatment earlier in the course of their illness, before they develop severe opportunistic infections.

Anemia was a strong predictor of mortality in our study. Patients with severe anemia had nearly 15 times higher risk of dying during the first year on ART compared to those with a normal hemoglobin level. Several studies from Europe and North America have shown that anemia is an independent predictor of mortality in patients on ART, even after controlling for CD4 cell count and viral load [22-24]. Recently, studies from developing countries have found the same association [9,13]. Indeed, in the largest African cohort study published to date, severe anemia (hemoglobin <8 g/dL) was the strongest independent predictor of mortality in 16 198 patients receiving ART in Zambia [13].

It is uncertain whether the association between anemia and mortality is causal or whether anemia is rather a marker of progressive HIV disease. It is known that the incidence of anemia increases with progression of HIV infection [23]. Furthermore, anemia can be a feature of certain opportunistic diseases, like disseminated mycobacterial infection and parvovirus B19 [25]. Several other etiologic factors may be involved in the development of HIV-associated anemia, including micronutrient deficiencies, immunological myelosuppression, impaired erythropoietin production and blood loss from intestinal opportunistic disease [25]. The role of iron supplementation is controversial, as some reports have suggested adverse effects of iron excess in HIV-infected individuals in industrialized countries [26,27]. On the contrary, recovery from anemia after erythropoietin treatment has been associated with improved survival [23,24], but high costs limit its use in poor countries. More recently, ART has been shown to significantly reduce HIV-associated anemia in developed countries [28,29]; however, this has not yet been investigated in rural Africa. Further studies are needed to explore possible interventions against HIV-associated anemia in resource-limited settings, including the role of iron supplementation.

Malnutrition was another strong, independent predictor of mortality in our study. Estimated one year mortality was nearly 50% among patients with severe malnutrition. Previously, studies from industrialized countries have shown that malnutrition in HIV infection is associated with morbidity and mortality, even after the introduction of highly active antiretroviral therapy in the late 1990s [30-32]. More recently, studies from developing countries have found that malnutrition is an independent predictor of mortality in patients starting ART [8,12,13,33]. However, it is not clear whether targeted therapy for malnutrition will result in improved survival [34]. Studies of nutritional interventions in HIV patients are urgently needed in developing countries, where malnutrition is often a result of poverty and food insecurity.

We found a reduced risk of death in patients starting ART in later calendar years compared with the initial period 2003–04. A possible explanation is that many patients with severe AIDS were included in the initial period, as this was the first clinic to offer ART in the area. However, since the risk reduction persisted after controlling for clinical stage, we believe that it may also be attributed to improved skills among local staff managing HIV patients. The decline in mortality over time supports our experience that non-physician clinicians can be trained to follow-up and treat HIV-infected patients.

To our knowledge, thrombocytopenia has never previously been shown to predict mortality in African patients on ART, although a few studies from North America have described an increased risk of disease progression and death [35,36]. Further research is needed to confirm our findings. WHO stage IV was not significantly associated with mortality in our study, in contrast to previous reports [1,8,11-14]. However, the comparison group was almost entirely composed of WHO stage III patients, which would weaken the statistical effect of WHO stage IV on mortality. Furthermore, the accuracy of clinical staging is probably quite variable in rural Africa. It is interesting that simple and more objective indicators identified in the present study appear to have a better predictive ability than clinical stage.

A prognostic model based on hemoglobin level had a strong predictive power in our study, separating the patients into low, low intermediate, high intermediate and high risk groups (Figure 2). Previously, similar survival curves for hemoglobin levels have been reported in European HIV patients, although anemia occurred less frequently [22]. Hemoglobin is a simple and inexpensive laboratory test, which can be performed even in rural, basic clinics. We believe it can be used as a simple and practical tool for initial risk assessment in the absence of CD4 cell count and viral load. Such early prognostic information would allow a more targeted search for opportunistic infections and closer follow-up in high-risk individuals, thus reducing excess mortality. Although the exact mortality figures from the present study can not necessarily be applied to other populations, we believe the concept of using hemoglobin level to identify patients with a poor prognosis can be used elsewhere. This simple prognostic model should be tested out in other African settings to assess its generalizability.

There are some weaknesses of our study. First, mortality might be underestimated, since patients lost to follow-up probably include individuals dying at home without being reported. Although the proportion of patients lost to follow-up in the present study (9.7%) was comparable to other African studies [12,13], data quality would be improved with better cohort retention. Second, the results might be affected by selection bias towards patients with more severe disease, since the study was conducted in a hospital setting. Third, some patients measured baseline hemoglobin shortly after ART initiation, which might have led to an overestimation of the prevalence of anemia in patients with a ZDV-based regimen. However, post-ART hemoglobin was only employed in a small number of patients, and it is unlikely that this has introduced any systematic bias into the study. Fourth, it is known that the generalizability of a prognostic system can be impaired if important independent predictors are left out [37]. We lacked reliable CD4 cell counts and viral loads, which are established predictors of morbidity and mortality in patients on ART [1]. However, our results strongly suggest that simple and available measurements can be useful alternative prognostic markers.

The main strength of our study is that it was carried out in a rural African hospital with use of national staff and inclusion of all eligible patients. Most other African ART studies have been performed in urban areas [9-11,13-15], in research settings with strict inclusion and exclusion criteria [38], or with support from an international non-governmental organization [8,10,12]. We believe that our results better reflect the reality in a rural hospital in sub-Saharan Africa, and thus may be applicable to other similar settings.

## Conclusion

We found high mortality among patients starting ART in this rural Tanzanian hospital, with the majority of deaths occurring within 3 months of ART initiation. Many patients enrolled with advanced immunodeficiency, and priority should be given to identify HIV-infected individuals and start ART earlier in the course of their illness. Anemia, thrombocytopenia and severe malnutrition were strong independent predictors of mortality. A simple prognostic model based on hemoglobin level appears to be a useful tool for initial risk assessment in resource-limited settings.

## Competing interests

The author(s) declares that they have no competing interests.

## Authors' contributions

AJ analyzed the data and drafted the manuscript. EN and BJN collected the data. LS performed the statistical analysis and helped to draft the manuscript. MIM participated in the conception of the study. HEA participated in the data collection and design of the study. SGG and JNB conceived the study, and participated in its design and coordination. All authors read and approved the final manuscript.

## Pre-publication history

The pre-publication history for this paper can be accessed here:


